# Predictors of mercury, lead, cadmium and antimony status in Norwegian never-pregnant women of fertile age

**DOI:** 10.1371/journal.pone.0189169

**Published:** 2017-12-05

**Authors:** Christina Herland Fløtre, Kristin Varsi, Thea Helm, Bjørn Bolann, Anne-Lise Bjørke-Monsen

**Affiliations:** 1 Faculty of Medicine, University of Bergen, Bergen, Norway; 2 Laboratory of Clinical Biochemistry, Haukeland University Hospital, Bergen, Norway; 3 Department of Clinical Science, Faculty of Medicine and Dentistry, University of Bergen, Bergen, Norway; Laurentian University, CANADA

## Abstract

**Background:**

The toxic trace elements mercury (Hg), lead (Pb), cadmium (Cd) and antimony (Sb) are transferred over the placenta to the fetus and secreted into the breastmilk. All four elements bioaccumulate in the body and as maternal age at delivery is increasing in industrialized countries, the burden of toxic trace elements in never-pregnant women of fertile age is of concern.

**Methods:**

Healthy, never-pregnant women aged 18 to 40 years (n = 158) were recruited between June 2012 and March 2015 in Bergen, Norway. Clinical data were collected and non-fasting venous blood samples were analyzed for whole blood Hg, Pb and Cd and serum Sb by ICP-MS and related to diet and life style factors.

**Results:**

In a multiple linear regression model, increasing age was associated with higher levels of Hg and Sb, but diet and life style factors were more important predictors. Median whole blood Hg was increased by a factor of 70 in women who had fish for dinner ≥1/week, compared to women who rarely or never ate fish (p<0.001). Alcohol intake was the strongest predictor for whole blood Pb, while use of tobacco was the strongest predictor for whole blood Cd. Being a vegetarian was associated with lower levels of both Hg and Sb.

**Conclusions:**

As toxic trace elements tend to bioaccumulate in the body, increasing maternal age at delivery may represent a threat to the next generation. In a group of healthy Norwegian never-pregnant women, age contributed to Hg and Sb levels, but diet and life style factors were stronger determinants of whole blood Hg, Pb, Cd and serum Sb levels. Continuous public actions are needed to reduce modifiable and preventable sources of potentially deleterious toxins to minimize the exposure in children and fertile women.

## Introduction

Mercury (Hg), cadmium (Cd), lead (Pb) and antimony (Sb) are toxic trace elements associated with negative health effects on the nervous, renal, cardiovascular and immune system [1–3]. Element interactions may also amplify the effect of even low dose concentrations [4]. The half-lives of the elements vary from 3 months for Hg [5], 2.7 years for Sb [6], while Pb and Cd have half-lives of about 30 years, so body stores tend to increase with age [7, 8].

As Hg and Pb and to a lesser degrees also Cd and Sb are transferred over the placenta to the fetus [9, 10] and after birth secreted into the breastmilk [11, 12], pregnancy represents a detoxification process for the mother, and higher parity is associated with lower levels of Hg, Pb and Cd [13]. Exposure to heavy metals during fetal life and infancy is associated with impairment of growth and central nervous system development, as well as pulmonary and nephrotic damage, and may have serious long-term health consequences for the child [14–17]. There are limited data on fetal exposure of Sb, but higher levels of Sb are reported in cord blood of pregnancies with an adverse outcome [18], but in general there are limited data on fetal and infant exposure of Sb.

Mean maternal age at delivery has increased during the last years and primiparas above 35 years are becoming increasingly common in industrialized countries [19]. A higher maternal age is associated with higher maternal morbidity, pregnancy complications [20], and later disease in the child [21]. Since 1980 there has been an increasing prevalence of autism specter disorders [22], a mental dysfunction that has been associated with advanced maternal age [23] and also higher levels of Hg, Pb, Cd and Sb in the children with autism specter disease compared to normal children [24].

As the age for giving birth to the first child is increasing, the burden of toxic elements in never-pregnant women of fertile age is of interest. We have evaluated the Hg, Pb, Cd and Sb status in healthy, never-pregnant Norwegian women aged of fertile age (18–40 years) and related this to age and life style factors associated with toxic trace element exposure [25–30].

## Materials and methods

### Study population and design

Between June 2012 and March 2015, healthy, never-pregnant women aged 18 to 40 years were recruited among employees and students at Haukeland University Hospital and the University of Bergen, Norway. In addition, healthy never-pregnant women in the same age-group with a vegetarian diet were recruited through Facebook, and complied with Facebook.com’s terms of service for recruiting participants on their website (www.facebook.com/policies/ads/).

Ethical approval of the protocol was granted by the Regional Committee of the Norwegian National Research Ethics Committee for Medical and Health Research (NEM) (2011/2447), and written informed consent was obtained from all women before enrollment.

The women completed a questionnaire concerning age, years of completed education, height, weight, use of medications, including oral contraceptives and hormone implants, diet, intake of alcohol, regular use of tobacco, including cigarettes and snuff, use of multiple micronutrient supplements (MMN), including vitamins, minerals, omega-3 fatty acids and cod oil. Use of supplements more than three days per week was defined as a regular user.

### Blood sampling and analyses

Non-fasting blood samples were obtained by antecubital venipuncture and collected into vacutainer tubes with EDTA and without additives approved for trace metal analysis (Terumo®, US). Five-hundred μL of whole blood and serum was aliquoted and stored at –80°C until analysis. Whole blood Hg, Pb and Cd and serum Sb were analyzed by ICP-MS on Perkin Elmer DRC-e (Perkin Elmer®, USA) in standard mode [31].

Plasma levels of cotinine were assayed using a LC-MS/MS method [32]. A plasma cotinine level ≥85 nmol/L is commonly used as a cut off to define regular smokers [33], however use of smokeless tobacco is associated with similar cotinine levels [34].

### Statistical analysis

Results are presented as mean and standard deviation (SD), compared by Student’s t-test or Anova, and median and interquartile range (IQR), compared by Mann-Whitney U test or Kruskal Wallis test. Chi-square test was used for categorical data. Spearman correlations and multiple linear regression models were used to explore relationships between data.

Graphical illustration of the relationship between whole blood Pb and alcohol units consumed per week was obtained by generalized additive models (GAM).

The SPSS statistical program (version 23) and the packages “mgcv” inR®, version 3.3 (The R Foundation for Statistical Computing) were used for the statistical analyses. Two-sided p-values < 0.05 were considered statistically significant.

## Results

### Demographics, nutrition and life style factors

A total of 158 healthy, never-pregnant women with an age range of 18 to 40 years, were included in the study. The majority 122/158 (78%) had a normal BMI (18.5 to 25.0), 6/158 (4%) were underweight (BMI<18.5), 24/158 (15%) were overweight (BMI: 25.0 to <30.0) and four women (3%) were obese (BMI≥30.0).

Most women, 124/158 (78%), had an omnivore diet, whereas 34/158 (22%) had used a vegetarian diet for a median of 36 months (IQR 16, 132), range 2–240 months. Fifteen of the 34 vegetarians (44%) were vegans and did not eat any animal products, including eggs and milk. Demographic data according to an omnivore or a vegetarian diet are given in Table 1.

**Table 1:** Baseline characteristics of healthy, never-pregnant women according to diet (n = 158).

		Omnivore diet N = 124	Vegetarian diet N = 34	P value
Age, years, mean (SD)		25.3 (4.8)	31.5 (4.3)	<0.001^a^
BMI, kg/m^2^, median (IQR)		21.7 (20.5, 23.7)	22.1 (20.7, 23.4)	0.88^b^
Education, n (%)				
	≤12 years	5 (4)	9 (27)	<0.001^c^
	13–17 years	32 (26)	18 (53)	
	≥17 years	87 (70)	7 (21)	
Regular users of supplements, (≥3 days/week), n (%)				
	Omega 3 fatty acids	59 (48)	11 (32)	0.11^c^
	Multivitamins/minerals	27 (22)	8 (24)	0.85^c^
Alcohol, number of units/week, median (IQR)		2.0 (0.5, 4.0)	2.0 (0.8, 3.0)	0.99^b^
Smokers, based on plasma cotinine≥85 nmol/L, n (%)		8 (7)	9 (27)	0.001^c^

^a^Comparison by Student's t-test

^b^Comparison by Mann-Whitney U test

^c^Comparison by Pearson Chi-square test

Omnivorous were younger, had a higher educational level and were less likely to use tobacco compared to vegetarians (Table 1). A total of four women (2.5%) reported regular daily smoking, two from each diet group, however, based on plasma cotinine levels ≥85 nmol/L, 17/158 (11%) women were defined as regular tobacco users. Use of alcohol ranged from 0 to 12 units per week, with no difference between the groups (Table 1). Two thirds (105/158) of the women reported regular use of micronutrient supplements, with no difference between omnivores and vegetarians (p>0.07) (Table 1).

Among the omnivores, the majority 86/124 (69%) had fish for dinner ≥1/week, while 38/158 (24%) rarely or never ate fish. The majority of the regular tobacco-users (10/17, 59%) rarely or never ate fish compared to non-users (28/140, 20%, p = 0.002). The most frequently eaten fish was farmed salmon (72%), followed by lean fish (17%) and other types of fatty fish (11%).

### Toxic trace element levels according to age, diet and life style factors

Levels of Hg, Pb, Cd and Sb according to age, diet, use of alcohol and tobacco are given in Table 2. Age was positively correlated to Hg (r = 0.18, p = 0.02), Cd (r = 0.27, p = 0.001) and Sb (r = 0.23, p = 0.004) by Spearman correlation, but only Cd and Sb levels were higher in women ≥25 years, when compared by Mann-Whitney to the younger age-group (Table 2).

**Table 2:** Levels of toxic trace elements according to age, diet, intake of alcohol and use of tobacco in healthy never-pregnant women (n = 158).

Parameters^a^	Age, years			Diet			Intake of alcohol, units per week			Use of tobacco determined by cotinine level		
	18–24 N = 87	25–40 N = 71	P value	Omnivore N = 124	Vegetarian N = 34	P value	<2 N = 65	≥2 N = 81	P value	<85 nmol/L N = 140	≥85 nmol/L N = 17	P value
Whole blood mercury, nmol/L	4.31 (1.55, 7.73)	4.92 (2.41, 9.19)	0.30	5.83 (3.85, 9.25)	0.13 (0, 0.93)	<0.001	4.85 (1.85, 8.70)	4.67 (1.73, 8.20)	0.93	4.93 (2.32, 8.82)	1.17 (0, 6.72)	0.04
Whole blood lead, μmol/L	0.04 (0.03, 0.05)	0.04 (0.03, 0.05)	0.62	0.04 (0.03, 0.05)	0.04 (0.03, 0.06)	0.30	0.03 (0.03, 0.05)	0.04 (0.04, 0.06)	<0.001	0.04 (0.03, 0.05)	0.05 (0.03, 0.06)	0.14
Whole blood cadmium, nmol/L	1.43 (1.11, 1.88)	1.88 (1.25, 2.59)	0.008	1.50 (1.15, 2.17)	1.70 (1.23, 2.57)	0.15	1.51 (1.14, 2.32)	1.51 (1.19, 1.98)	0.56	1.51 (1.14, 2.10)	2.47 (1.50, 7.66)	0.009
Serum antimony, nmol/L	28.7 (26.3, 31.8)	31.2 (28.7, 33.6)	0.002	30.2 (27.3, 33.6)	29.2 (26.4, 31.0)	0.09	31.2 (28.0, 33.6)	29.5 (27.0, 32.7)	0.06	30.0 (27.0, 32.7)	30.6 (27.6, 34.1)	0.24

^a^Median and IQR, compared by Mann-Whitney test

Age remained a significant predictor for Hg and Sb in a multiple linear regression model, which additionally included type of diet, fish intake, alcohol intake, use of tobacco, education, BMI and use of omega 3/cod oil supplements (Table 3).

**Table 3:** Determinants of toxic trace elements levels in healthy never-pregnant women (n = 158) by multiple linear regression.

Variables included in the model	Whole blood mercury, nmol/L		Whole blood lead, μmol/L		Whole blood cadmium, nmol/L		Serum antimony, nmol/L	
	*Beta*	P	*Beta*	P	*Beta*	P	*Beta*	P
Age^a^	0.18	0.03	0.10	0.22	0.08	0.32	0.19	0.02
Diet^b^	-0.04	0.80	0.09	0.53	0.03	0.86	-0.37	0.01
Fish consumption^c^	0.45	0.002	-0.01	0.95	0.15	0.26	-0.24	0.10
Alcohol intake^d^	0.04	0.66	0.37	<0.001	0.16	0.048	-0.06	0.51
Regular use of tobacco^e^	-0.04	0.63	0.04	0.63	0.45	<0.001	0.19	0.03
Education^f^	-0.13	0.13	0.10	0.28	-0.20	0.02	-0.04	0.69

BMI and use of omega 3 supplements/cod oil were additionally included in the model

^a^ Age in years

^b^ Omnivore versus vegetarian diet

^c^ Fish consumption categorized; Fish for dinner:<1/month, 1-3/month or ≥1/week

^d^ Intake of alcohol units/week

^e^ Based on plasma cotinine levels, categorized; <85 nmol/L, ≥85 nmol/L

^f^ Education, categorized; ≤12 years, 13–17 years, ≥17 years.

Vegetarians had lower levels of Hg and Sb compared to omnivores (Table 2), and this was evident also in the multiple linear regression model (Table 3). No differences in levels of toxic trace elements were seen between vegans and vegetarians (p>0.22, data not shown).

Fish intake was the strongest predictor for Hg in the multiple linear regression model (Table 3). Median whole blood Hg was increased by a factor of 70 in women who had fish for dinner ≥ 1/week, compared to women who rarely or never ate fish (Table 4).

**Table 4:** Levels of toxic trace elements according to fish intake in healthy never-pregnant women (n = 158).

Toxic trace elements^a^	Consumption of fish for dinner			P value
	<1 meal/month N = 38	1–3 meals/month N = 34	≥1 meal/week N = 86	
Whole blood mercury, nmol/L	0.1 (0, 1.1)	4.4 (2.5, 7.1)	7.0 (4.3, 11.0)	<0.001
Whole blood lead, μmol/L	0.04 (0.03, 0.06)	0.04 (0.03, 0.05)	0.04 (0.03, 0.05)	0.73
Whole blood cadmium, nmol/L	1.8 (1.2, 2.4)	1.4 (1.1, 2.0)	1.5 (1.2, 2.2)	0.27
Serum antimony, nmol/L	29.4 (27.0, 31.3)	31.2 (27.5, 33.8)	30.0 (27.0, 32.9)	0.33

^a^ Median and (IQR) **c**ompared by Kruskall-Wallis test

**Fig 1:**
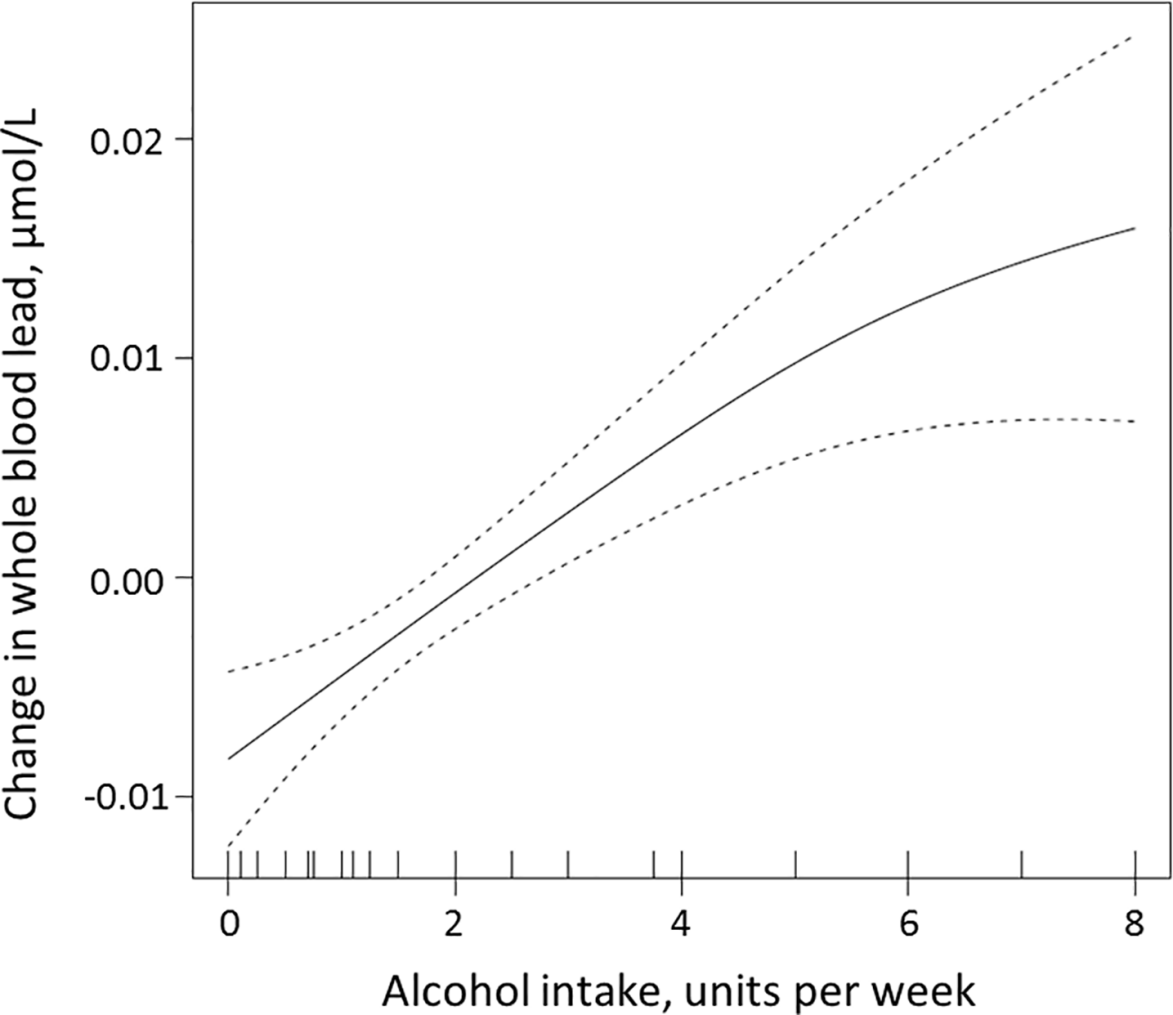
The association of alcohol intake in units per week with Pb levels by generalized additive model (GAM), adjusted for age. The solid line shows the fitted model and the area between the dotted lines indicate 95%.

Alcohol intake was correlated to Pb by Spearman correlation (r = 0.37, p<0.001), and median Pb was significantly higher in women who drank 2 or more units of alcohol per week (Table 2). There was a dose-response relationship between intake of alcohol in units per week and Pb, as shown by GAM corrected for age (Fig 1). Alcohol was also the only significant predictor for Pb in the multiple linear regression model (Table 3).

Regular use of tobacco resulting in plasma cotinine levels ≥85 μmol/L was associated with higher Cd and lower Hg levels (Table 2). In the multiple linear regression models, tobacco use was strongly related to Cd and less to Sb.

A longer education was a negative predictor for Cd, while no significant associations to any of the trace elements were seen for BMI or use of omega 3 fatty acids/ cod oil supplements by multiple linear regression models (Table 3).

## Discussion

In this group of never-pregnant women of fertile age, age was associated with higher levels of Hg and Sb, but the strongest predictor for Hg status was fish intake, alcohol intake for Pb, use of tobacco for Cd status, while the strongest predictor for Sb was an omnivore diet.

### Strength and limitations

This was an observational study with self-reported clinical data, known to have disadvantages, as highlighted by the discrepancy between reported smoking status and plasma cotinine levels. However, as we were able to measure plasma cotinine this problem was diminished. This study included women of fertile age, ranging from 18 to 40 years, however, the majority of our population was below 30 years, which disabled us to fully investigate the effect of a more advanced age on toxic trace element status.

### An omnivore versus a vegetarian diet

Published data concerning toxic element status in vegetarians compared to omnivores are somewhat conflicting [28, 35, 36]. In a non-smoking population, vegetarians had higher levels of Cd compared to omnivores [28], and both vegan and vegetarian diets have been proposed to be a risk factor for increasing the level of several toxic trace elements in the body [35]. However, in a Swedish study, a change from a mixed to a lactovegetarian diet for 3 months was associated with lower hair concentrations of mercury, lead, and cadmium [36].

We did not find any difference in Pb and Cd according to diet. An omnivore diet was a predictor for Sb, and the negligible Hg levels we found in our vegetarian population compared to omnivores with a high fish intake, confirms the notion that Hg levels are primarily the result of intake of fish and seafood [27] and therefore reduced in vegetarians.

### Mercury

The level of Hg is reported to increase with age [37], confirmed by our data on women aged 18 to 40 years. Compared to published studies (Table 5), the median Hg value (4.91 nmol/L) in our population was low. Hg levels vary in different populations according to published studies [37–43] (Table 5). A considerable higher Hg level, geometrical mean (GM) 13.1 nmol/L, was reported in Korean women above 20 years in 2008 [41], while a lower level, median 3.74 nmol/L, was found in a Czech mixed population aged 18–58 years in 2009 [39] (Table 5).

**Table 5:** Concentrations of whole blood mercury, lead, cadmium and serum antimony in different populations.

Value	Statistical	Number	Year	Sex	Age group	Nationality	Reference
**Whole blood mercury, nmol/L**							
13.1 (12.3, 13.8)	GM (95% CI)	3531	2008	Female	>20	Korean	[41]
11.47 (1.1, 3.5)	P95 (95% CI)	1229	2012–2013	Mixed	20–79	Canadian	[44]
9.22 (8.43, 10.02)	Mean (95% CI)	123	1997	Female	31	Finnish	[38]
7.98	Median	30	2013	Mixed	Adults < 40 years	Norwegian	[37]
7.93 (3.44, 25.92)	GM (10–95 percentile)	73	2011–2012	Female	18–40	Danish	[43]
6.88 (6.58, 7.23)	Mean (95% CI)	1992	2008–2010	Mixed	20–59	French	[42]
4.91 (0, 19.34)	Median (2.5, 97.5 percentile)	158	2011–2015	Female	18–39	Norwegian	Fløtre et al
4.69	GM	185	2006	Women	18–39	Brazilian	[40]
3.74	Median	1227	2009	Mixed	18–58	Czech	[39]
**Whole blood lead, μmol/L**							
0.16 (0.15, 0.17)	P95 (95% CI)	3142	2012–2013	Mixed	20–79	Canadian	[44]
0.09 P95 (95% CI)	GM	1992	2008–2010	Mixed	20–59	French	[42]
0.09	Median	30	2013	Mixed	Adults < 40 years	Norwegian	[37]
0.08	GM	158	2006	Female	18–39	Brazilian	[40]
0.07	Median	494	2009	Female	18–58	Czech	[39]
0.05	GM	2892	2007–2010	Mixed	20–39	American	[45]
0.04 (0.02, 0.11)	Median (2.5, 97.5 percentile)	158	2011–2015	Female	18–39	Norwegian	Fløtre et al
0.04 (0.03, 0.08)	GM (10–95 percentile)	73	2011–2012	Female	18–40	Danish	[43]
0.04 (0.04, 0.05)	GM (95% CI)	123	1997	Female	31	Finnish	[38]
**Whole blood cadmium, nmol/L**							
7.38 (6.40, 8.45)	P95 (95% CI)	2507	2012–2013	Mixed	20–79	Canadian	[44]
3.47 (3.38, 3.65)	GM (95% CI)	1992	2008–2010	Mixed	20–59	French	[42]
2.94	Median	30	2013	Mixed	Adults < 40 years	Norwegian	[37]
2.76	GM	2892	2007–2010	Mixed	20–39	American	[45]
2.67	Median	896	2009	Mixed	18–58 years, non-smokers	Czech	[39]
1.78 (0.63, 6.76]	GM (10–95 percentile)	73	2011–2012	Female	18–40	Danish	[43]
1.53 (0.68, 7.75)	Median (2.5, 97.5 percentile)	158	2011–2015	Female	18–39	Norwegian	Fløtre et al
1.07 (0.89–1.25)	GM (95% CI)	123	1997	Females	31	Finnish	[38]
0.9	GM	185	2006	Females	18–39	Brazilian	[40]
**Serum antimony, nmol/L**							
30.1 (21.2, 38.7)	Median (2.5, 97.5 percentile)	158	2011–2015	Female	18–39	Norwegian	Fløtre et al
14.78 ± 8.9	GM ± SD	24	2014	Female	23–64	Chinese	[46]

The high level of blood mercury in the Korean study has been explained by a high seafood consumption in this population [41]. Fish consumption is generally high in Norway [47], but the lower median Hg level in our population might be due to a high percentage of vegetarians (22%). Omnivores with a high fish intake had a median Hg level of 7.0 nmol/L, comparable to Danish women of fertile age (geometrical mean (GM) 7.93 nmol/L) [43] (Table 5). Tobacco-users had also lower levels of Hg and conversely a lower intake of fish, compared to non-users of tobacco. Smoking is associated with overall unhealthy behavior, including poor nutrition [48], while eating fish and seafood is considered to be beneficial [49]. According to current Nordic recommendations, Norwegians should have an intake of 300–450 g fish per week, of which at least 200 g should be fatty fish, with no discrimination according to age and sex [50]. Fish provides important nutrients, it is however important to consider the fact that fish is also the most important contributor of Hg, known to have serious negative health effects, even in low doses [3].

### Lead

The median Pb value of 0.04 μmol/L was low in our population and comparable to published data from Nordic female populations [38, 41, 43, 44] (Table 5). Pb levels in premenopausal, non-pregnant women are primarily due to exposures to contaminated food or water [51]. Pb are commonly found in brewed tea [52], reported to be a popular drink among women [53], however, as we did not have any data on tea consumption, we were unable to evaluate this factor. Pb is also commonly found in red wine [54], which corroborate with our finding of alcohol intake being the only significant predictor for Pb level. According to 2014 data from the Norwegian Institute of Public Health, the alcohol consume has increased by approximately 40% in Norway during the last 20 years, particularly among women, who preferably drink wine [55].

### Cadmium

We found a median Cd value of 1.53 nmol/L, which is low compared to other countries (Table 5). As smoking has been associated with higher levels of Cd [25], this finding might be related to a low prevalence of smokers in our population. The use of snuff is becoming increasingly popular in Norway [56], but studies on how use of smokeless tobacco affect toxic element status are difficult to find [57]. We found a 63% higher median Cd level in regular users of tobacco, confirmed by cotinine levels, compared to non-users. The median cadmium level in our non-users of tobacco, 1.51 nmol/L, was about twice as high as the value of Finnish non-smokers aged 31 years, 0.62 nmol/L, while the median level in our current tobacco-users, 2.47 nmol/L, was substantially lower than in the Finnish smokers, 6.04 nmol/L [38].

### Antimony

Our median Sb levels (30.1 nmol/L) was comparable, but higher than in Chinese females aged 23–64 years in a study from 2014,where they found a geometric mean (SD) of 14.78 (± 8.9) nmol/L [46] (Table 5). The most significant predictor for Sb in our population was an omnivore diet, followed by age and use of tobacco. Sb has been found in all food groups, with the highest concentrations in dairy products [26]. Sb levels are also reported to be higher in smokers [58], as confirmed by our results.

Occupational exposure in electronic waste recycling was the most important source of Sb in the Chinese population, while dietary exposure was considered to be low [58]. Data on environmental exposure sources of Sb is scarce and further studies are obviously needed. Sb is frequently found in mobile phones [58], a popular electronic article, however, whether the use of mobile phones may contribute to higher serum levels is unknown.

Sb has been reported to cause neurotoxicity, liver and respiratory disease and has been associated with an increased cardiovascular risk [1, 10]. Sb may cross the placenta [1], and is also found in breastmilk [11]. It has been suggested that antimony trioxide may interfere with fetal development [1, 59] and higher concentrations of Sb have been found in cord blood in pregnancies with an adverse outcome [18].

### Implications

Element interactions may amplify the effect of even low dose concentrations of toxic elements [4] and safe limits are considered to be unlikely [60]. As toxic elements tend to bioaccumulate in the body [37, 61], a higher maternal age may have an impact on the burden of environmental toxins transferred to the next generation. However, we find that fish intake, alcohol consumption and use of tobacco, are more important determinants for Hg, Pb and Cd status than age in females from 18 to 40 years.

As the only possible way to protect a fetus is to minimize the exposure of toxic elements in fertile women [62], continuous public actions are needed for reducing these modifiable and preventable sources of potentially deleterious toxins.

## Conclusion

As toxic trace elements tend to bioaccumulate in the body, increasing maternal age at delivery may represent a threat to the next generation. In this study of Norwegian never-pregnant women aged 18 to 40 years, older age was associated with higher levels of Hg and Sb, but the main predictor for Hg levels was fish intake, for Pb alcohol intake, for Cd regular use of tobacco, while an omnivorous diet was the strongest predictor for Sb levels. As these are modifiable and preventable sources of harmful toxic elements in fertile women, public consideration and actions are needed to minimize the potential transfer of toxins to the next generation.

## Supporting information

S1 Data Fløtre et all data, containing the data for the study. (XLSX)

## References

[1] Cooper RG, Harrison AP. The exposure to and health effects of antimony. Ind J Occup Environ Med. 2009;13(1):3–10. 10.4103/0019-5278.50716 PMC2822166 20165605

[2] Jarup L. Hazards of heavy metal contamination. Br Med Bull. 2003;68:167–82. 14757716 10.1093/bmb/ldg032

[3] Zahir F, Rizwi SJ, Haq SK, Khan RH. Low dose mercury toxicity and human health. Environ Toxicol Pharmacol. 2005;20(2):351–60. doi: 10.1016/j.etap.2005.03.007 21783611 10.1016/j.etap.2005.03.007

[4] Cobbina SJ, Chen Y, Zhou Z, Wu X, Feng W, Wang W, et al Low concentration toxic metal mixture interactions: Effects on essential and non-essential metals in brain, liver, and kidneys of mice on sub-chronic exposure. Chemosphere. 2015;132:79–86. doi: 10.1016/j.chemosphere.2015.03.013 25828250 10.1016/j.chemosphere.2015.03.013

[5] Yaginuma-Sakurai K, Murata K, Iwai-Shimada M, Nakai K, Kurokawa N, Tatsuta N, et al Hair-to-blood ratio and biological half-life of mercury: experimental study of methylmercury exposure through fish consumption in humans. J Toxicol Sci. 2012;37(1):123–30. 22293416 10.2131/jts.37.123

[6] De Boeck M, Kirsch-Volders M, Lison D. Cobalt and antimony: genotoxicity and carcinogenicity. Mutat Res. 2003;533(1–2):135–52. 14643417 10.1016/j.mrfmmm.2003.07.012

[7] Abadin H, Ashizawa A, Stevens YW, Llados F, Diamond G, Sage G, et al Toxicological Profile for Lead. Agency for Toxic Substances and Disease Registry (ATSDR) Toxicological Profiles Atlanta (GA) 2007. 24049859

[8] Jarup L, Akesson A. Current status of cadmium as an environmental health problem. Toxicol Appl Pharm. 2009;238(3):201–8. 10.1016/j.taap.2009.04.020 19409405

[9] Arbuckle TE, Liang CL, Morisset AS, Fisher M, Weiler H, Cirtiu CM, et al Maternal and fetal exposure to cadmium, lead, manganese and mercury: The MIREC study. Chemosphere. 2016;163:270–82. doi: 10.1016/j.chemosphere.2016.08.023 27540762 10.1016/j.chemosphere.2016.08.023

[10] Barbieri FL, Gardon J, Ruiz-Castell M, Paco VP, Muckelbauer R, Casiot C, et al Toxic trace elements in maternal and cord blood and social determinants in a Bolivian mining city. Int J Environ Health Res. 2016;26(2):158–74. doi: 10.1080/09603123.2015.1061114 26179629 10.1080/09603123.2015.1061114 PMC4733940

[11] Iyengar GV, Kasperek K, Feinendegen LE, Wang YX, Weese H. Determination of Co, Cu, Fe, Hg, Mn, Sb, Se and Zn in Milk Samples. Sci Total Environ. 1982;24(3):267–74. 7123209 10.1016/0048-9697(82)90005-5

[12] Rebelo FM, Caldas ED. Arsenic, lead, mercury and cadmium: Toxicity, levels in breast milk and the risks for breastfed infants. Environ Res. 2016;151:671–88. doi: 10.1016/j.envres.2016.08.027 27619212 10.1016/j.envres.2016.08.027

[13] Jain RB. Effect of Pregnancy on the Levels of Blood Cadmium, Lead, and Mercury for Females Aged 17–39 Years Old: Data from National Health and Nutrition Examination Survey 2003–2010. J Toxicol Env Heal A. 2013;76(1):58–69. 10.1080/15287394.2012.722524 23151210

[14] Counter SA, Buchanan LH. Mercury exposure in children: a review. Toxicol Appl Pharm. 2004;198(2):209–30. 10.1016/j.taap.2003.11.032 15236954

[15] Rodrigues EG, Bellinger DC, Valeri L, Hasan MOSI, Quamruzzaman Q, Golam M, et al Neurodevelopmental outcomes among 2-to 3-year-old children in Bangladesh with elevated blood lead and exposure to arsenic and manganese in drinking water. Environ Health-Glob. 2016;15. 10.1186/s12940-016-0127-y PMC4788832 26968381

[16] Sanders AP, Claus Henn B, Wright RO. Perinatal and Childhood Exposure to Cadmium, Manganese, and Metal Mixtures and Effects on Cognition and Behavior: A Review of Recent Literature. Curr Environ Health Rep. 2015;2(3):284–94. doi: 10.1007/s40572-015-0058-8 26231505 10.1007/s40572-015-0058-8 PMC4531257

[17] Tang ML, Xu CY, Lin N, Liu K, Zhang YL, Yu XW, et al Lead, mercury, and cadmium in umbilical cord serum and birth outcomes in Chinese fish consumers. Chemosphere. 2016;148:270–5. doi: 10.1016/j.chemosphere.2016.01.058 26812370 10.1016/j.chemosphere.2016.01.058

[18] Zheng G, Zhong H, Guo Z, Wu Z, Zhang H, Wang C, et al Levels of heavy metals and trace elements in umbilical cord blood and the risk of adverse pregnancy outcomes: a population-based study. Biol Trace Elem Res. 2014;160(3):437–44. doi: 10.1007/s12011-014-0057-x 25008990 10.1007/s12011-014-0057-x

[19] Carolan M. The graying of the obstetric population: implications for the older mother. J Obstet Gynecol Neonatal Nurs. JOGNN. 2003;32(1):19–27. 12570178 10.1177/0884217502239797

[20] Cleary-Goldman J, Malone FD, Vidaver J, Ball RH, Nyberg DA, Comstock CH, et al Impact of maternal age on obstetric outcome. Obstet Gynecol. 2005;105(5 Pt 1):983–90. 15863534 10.1097/01.AOG.0000158118.75532.51

[21] Hviid MM, Skovlund CW, Morch LS, Lidegaard O. Maternal age and child morbidity: A Danish national cohort study. PloS One. 2017;12(4):e0174770 doi: 10.1371/journal.pone.0174770 28380000 10.1371/journal.pone.0174770 PMC5381873

[22] Newschaffer CJ, Croen LA, Daniels J, Giarelli E, Grether JK, Levy SE, et al The epidemiology of autism spectrum disorders. Annu Rev Public Health. 2007;28:235–58. doi: 10.1146/annurev.publhealth.28.021406.144007 17367287 10.1146/annurev.publhealth.28.021406.144007

[23] Lee BK, McGrath JJ. Advancing parental age and autism: multifactorial pathways. Trends Mol Med. 2015;21(2):118–25. doi: 10.1016/j.molmed.2014.11.005 25662027 10.1016/j.molmed.2014.11.005

[24] Saghazadeh A, Rezaei N. Systematic review and meta-analysis links autism and toxic metals and highlights the impact of country development status: Higher blood and erythrocyte levels for mercury and lead, and higher hair antimony, cadmium, lead, and mercury. Prog Neuropsychopharmacol Biol Psychiatry. 2017;79(Pt B):340–68. doi: 10.1016/j.pnpbp.2017.07.011 28716727 10.1016/j.pnpbp.2017.07.011

[25] Aoki Y, Yee J, Mortensen ME. Blood cadmium by race/hispanic origin: The role of smoking. Environ Res. 2017;155:193–8. doi: 10.1016/j.envres.2017.02.016 28231546 10.1016/j.envres.2017.02.016 PMC5615218

[26] Fu Z, Wu F, Mo C, Deng Q, Meng W, Giesy JP. Comparison of arsenic and antimony biogeochemical behavior in water, soil and tailings from Xikuangshan, China. Sci Total Environ. 2016;539:97–104. doi: 10.1016/j.scitotenv.2015.08.146 26356182 10.1016/j.scitotenv.2015.08.146

[27] Kim KH, Kabir E, Jahan SA. A review on the distribution of Hg in the environment and its human health impacts. J Hazard Mater. 2016;306:376–85. doi: 10.1016/j.jhazmat.2015.11.031 26826963 10.1016/j.jhazmat.2015.11.031

[28] Krajcovicova-Kudladkova M, Ursinyova M, Masanova V, Bederova A, Valachovicova M. Cadmium blood concentrations in relation to nutrition. Cent Eur J Public Health. 2006;14(3):126–9. 17152224 10.21101/cejph.a3385

[29] Mielke HW, Reagan PL. Soil is an important pathway of human lead exposure. Environ Health Perspect. 1998;106 Suppl 1:217–29. 10.1289/ehp.98106s1217 PMC1533263 9539015

[30] Satarug S, Vesey DA, Gobe GC. Current health risk assessment practice for dietary cadmium: Data from different countries. Food Chem Toxicol. 2017;106(Pt A):430–45. doi: 10.1016/j.fct.2017.06.013 28602857 10.1016/j.fct.2017.06.013

[31] Bolann BJ, Distante S, Morkrid L, Ulvik RJ. Bloodletting therapy in hemochromatosis: Does it affect trace element homeostasis? J Trace Elem Med Biol. 2015;31:225–9. doi: 10.1016/j.jtemb.2014.07.021 25175510 10.1016/j.jtemb.2014.07.021

[32] Midttun O, Kvalheim G, Ueland PM. High-throughput, low-volume, multianalyte quantification of plasma metabolites related to one-carbon metabolism using HPLC-MS/MS. Anal Bioanal Chem. 2013;405(6):2009–17. doi: 10.1007/s00216-012-6602-6 23232958 10.1007/s00216-012-6602-6

[33] Kim S. Overview of Cotinine Cutoff Values for Smoking Status Classification. Int J Environ Res Public Health. 2016;13(12). 10.3390/ijerph13121236 PMC5201377 27983665

[34] Gritz ER, Baer-Weiss V, Benowitz NL, Van Vunakis H, Jarvik ME. Plasma nicotine and cotinine concentrations in habitual smokeless tobacco users. Clin Pharmacol Ther. 1981;30(2):201–9. 7195786 10.1038/clpt.1981.149

[35] Rossi V RA, Pigatto PD, Barbaro M, Bolengo I, Guzzi G. Vegan diet and the risk of exposure to major toxic metals. Toxicol Lett. 2016;258(9 16):S109–S.

[36] Srikumar TS, Johansson GK, Ockerman PA, Gustafsson JA, Akesson B. Trace element status in healthy subjects switching from a mixed to a lactovegetarian diet for 12 mo. Am J Clin Nutr. 1992;55(4):885–90. 1550072 10.1093/ajcn/55.4.885

[37] Birgisdottir BE, Knutsen HK, Haugen M, Gjelstad IM, Jenssen MT, Ellingsen DG, et al Essential and toxic element concentrations in blood and urine and their associations with diet: results from a Norwegian population study including high-consumers of seafood and game. Sci Total Environ. 2013;463–464:836–44. doi: 10.1016/j.scitotenv.2013.06.078 23867847 10.1016/j.scitotenv.2013.06.078

[38] Abass K, Koiranen M, Mazej D, Tratnik JS, Horvat M, Hakkola J, et al Arsenic, cadmium, lead and mercury levels in blood of Finnish adults and their relation to diet, lifestyle habits and sociodemographic variables. Environ Sci Pollut Res Int. 2017;24(2):1347–62. doi: 10.1007/s11356-016-7824-5 27778267 10.1007/s11356-016-7824-5

[39] Cerna M, Krskova A, Cejchanova M, Spevackova V. Human biomonitoring in the Czech Republic: an overview. Int J Hyg Environ Health. 2012;215(2):109–19. doi: 10.1016/j.ijheh.2011.09.007 22014893 10.1016/j.ijheh.2011.09.007

[40] Kuno R, Roquetti MH, Becker K, Seiwert M, Gouveia N. Reference values for lead, cadmium and mercury in the blood of adults from the metropolitan area of Sao Paulo, Brazil. Int J Hyg Environ Health. 2013;216(3):243–9. doi: 10.1016/j.ijheh.2012.05.010 22748699 10.1016/j.ijheh.2012.05.010

[41] Lee JW, Lee CK, Moon CS, Choi IJ, Lee KJ, Yi SM, et al Korea National Survey for Environmental Pollutants in the Human Body 2008: heavy metals in the blood or urine of the Korean population. Int J Hyg Environ Health. 2012;215(4):449–57. doi: 10.1016/j.ijheh.2012.01.002 22341685 10.1016/j.ijheh.2012.01.002

[42] Nisse C, Tagne-Fotso R, Howsam M, Members of Health Examination Centres of the Nord—Pas-de-Calais region n, Richeval C, Labat L, et al Blood and urinary levels of metals and metalloids in the general adult population of Northern France: The IMEPOGE study, 2008–2010. Int J Hyg Environ Health. 2017;220(2 Pt B):341–63. 27931767 10.1016/j.ijheh.2016.09.020

[43] Rosofsky A, Janulewicz P, Thayer KA, McClean M, Wise LA, Calafat AM, et al Exposure to multiple chemicals in a cohort of reproductive-aged Danish women. Environ Res. 2017;154:73–85. doi: 10.1016/j.envres.2016.12.011 28039828 10.1016/j.envres.2016.12.011 PMC5328929

[44] Saravanabhavan G, Werry K, Walker M, Haines D, Malowany M, Khoury C. Human biomonitoring reference values for metals and trace elements in blood and urine derived from the Canadian Health Measures Survey 2007–2013. Int J Hyg Environ Health. 2017;220(2 Pt A):189–200. 27776932 10.1016/j.ijheh.2016.10.006

[45] Scinicariello F, Buser MC. Blood cadmium and depressive symptoms in young adults (aged 20–39 years). Psychol Med. 2015;45(4):807–15. doi: 10.1017/S0033291714001883 25115444 10.1017/S0033291714001883 PMC4571450

[46] Li J, Cen D, Huang D, Li X, Xu J, Fu S, et al Detection and analysis of 12 heavy metals in blood and hair sample from a general population of Pearl River Delta area. Cell Biochem Biophys. 2014;70(3):1663–9. doi: 10.1007/s12013-014-0110-6 25009099 10.1007/s12013-014-0110-6

[47] Jenssen MT, Brantsaeter AL, Haugen M, Meltzer HM, Larssen T, Kvalem HE, et al Dietary mercury exposure in a population with a wide range of fish consumption—self-capture of fish and regional differences are important determinants of mercury in blood. Sci Total Environ. 2012;439:220–9. doi: 10.1016/j.scitotenv.2012.09.024 23069934 10.1016/j.scitotenv.2012.09.024

[48] Pampel FC, Krueger PM, Denney JT. Socioeconomic Disparities in Health Behaviors. Annu Rev Sociol. 2010;36:349–70. doi: 10.1146/annurev.soc.012809.102529 21909182 10.1146/annurev.soc.012809.102529 PMC3169799

[49] Hosomi R, Yoshida M, Fukunaga K. Seafood consumption and components for health. Glob J Health Sci. 2012;4(3):72–86. doi: 10.5539/gjhs.v4n3p72 22980234 10.5539/gjhs.v4n3p72 PMC4776937

[50] Hornell A, Lagstrom H, Lande B, Thorsdottir I. Breastfeeding, introduction of other foods and effects on health: a systematic literature review for the 5th Nordic Nutrition Recommendations. Food Nutr Res. 2013;57. 10.3402/fnr.v57i0.20823 PMC3625706 23589711

[51] Jackson LW, Howards PP, Wactawski-Wende J, Schisterman EF. The association between cadmium, lead and mercury blood levels and reproductive hormones among healthy, premenopausal women. Hum Reprod. 2011;26(10):2887–95. doi: 10.1093/humrep/der250 21778284 10.1093/humrep/der250 PMC3174033

[52] Schwalfenberg G, Genuis SJ, Rodushkin I. The benefits and risks of consuming brewed tea: beware of toxic element contamination. J Toxicol. 2013;2013:370460 doi: 10.1155/2013/370460 24260033 10.1155/2013/370460 PMC3821942

[53] Ek WE, Tobi EW, Ahsan M, Lampa E, Ponzi E, Kyrtopoulos SA, et al Tea and coffee consumption in relation to DNA methylation in four European cohorts. Hum Mol Gen. 2017. 10.1093/hmg/ddx194 PMC6455036 28535255

[54] Naughton DP, Petroczi A. Heavy metal ions in wines: meta-analysis of target hazard quotients reveal health risks. Chem Cent J. 2008;2:22 doi: 10.1186/1752-153X-2-22 18973648 10.1186/1752-153X-2-22 PMC2628338

[55] Christophersen A G, H., Nesvåg, R., Ystrøm, E. Alcohol and other psychoactive substances—Public Health Report NIPH, 2014.

[56] Kvaavik E, Lund I, Nygard M, Hansen BT. Lifestyle Correlates of Female Snus Use and Smoking: A Large Population-Based Survey of Women in Norway. Nicotine Tob Res. 2016;18(4):431–6. doi: 10.1093/ntr/ntv126 26069033 10.1093/ntr/ntv126

[57] Song MA, Marian C, Brasky TM, Reisinger S, Djordjevic M, Shields PG. Chemical and toxicological characteristics of conventional and low-TSNA moist snuff tobacco products. Toxicol Lett. 2016;245:68–77. doi: 10.1016/j.toxlet.2016.01.012 26802282 10.1016/j.toxlet.2016.01.012 PMC4910161

[58] Huang Y, Ni W, Chen Y, Wang X, Zhang J, Wu K. Levels and risk factors of antimony contamination in human hair from an electronic waste recycling area, Guiyu, China. Environmental science and pollution research international. 2015;22(9):7112–9. doi: 10.1007/s11356-014-3941-1 25501644 10.1007/s11356-014-3941-1

[59] Leonard A, Gerber GB. Mutagenicity, carcinogenicity and teratogenicity of antimony compounds. Mutat Res. 1996;366(1):1–8. 8921983 10.1016/s0165-1110(96)90003-2

[60] Tchounwou PB, Yedjou CG, Patlolla AK, Sutton DJ. Heavy metal toxicity and the environment. Exs. 2012;101:133–64. doi: 10.1007/978-3-7643-8340-4_6 22945569 10.1007/978-3-7643-8340-4_6 PMC4144270

[61] Fontaine J, Dewailly E, Benedetti JL, Pereg D, Ayotte P, Dery S. Re-evaluation of blood mercury, lead and cadmium concentrations in the Inuit population of Nunavik (Quebec): a cross-sectional study. Environ Health. 2008;7:25 doi: 10.1186/1476-069X-7-25 18518986 10.1186/1476-069X-7-25 PMC2442064

[62] Taylor CM, Golding J, Emond AM. Lead, cadmium and mercury levels in pregnancy: the need for international consensus on levels of concern. J Dev Orig Hlth Dis. 2014;5(1):16–30. 10.1017/S2040174413000500 24847687

